# Supplementary material for: Unveiling the Bioactive Potential of the Invasive Jellyfish Phyllorhiza punctata Through Integrative Transcriptomic and Proteomic Analyses
Source: Biomolecules. 2025 Aug 4;15(8):1121. doi: 10.3390/biom15081121 (PMC12383608; doi:10.3390/biom15081121)
Supplement: Supplementary file 1 [file biomolecules-15-01121-s001.zip › Suplementary Material/FileS3/PEPFOLD4/XP_065066105_5792/clusters.html]

 PEP-FOLD clusters


Download archive of all models  here   

|  |  |  |
| --- | --- | --- |
| # | file | sOPEP |
| # cluster 1 - Size: 6 |  |  |
|  | XP\_065066105\_5792-model1 | -22.3939 |
|  | XP\_065066105\_5792-model1.1 | -22.2437 |
|  | XP\_065066105\_5792-model1.2 | -22.0502 |
|  | XP\_065066105\_5792-model1.3 | -21.8956 |
|  | XP\_065066105\_5792-model1.4 | -21.8557 |
|  | XP\_065066105\_5792-model1.5 | -21.1984 |
| # cluster 2 - Size: 12 |  |  |
|  | XP\_065066105\_5792-model2 | -22.0885 |
|  | XP\_065066105\_5792-model2.1 | -21.904 |
|  | XP\_065066105\_5792-model2.2 | -21.8447 |
|  | XP\_065066105\_5792-model2.3 | -21.7186 |
|  | XP\_065066105\_5792-model2.4 | -21.5495 |
|  | XP\_065066105\_5792-model2.5 | -21.4785 |
|  | XP\_065066105\_5792-model2.6 | -21.4545 |
|  | XP\_065066105\_5792-model2.7 | -21.4077 |
|  | XP\_065066105\_5792-model2.8 | -21.2237 |
|  | XP\_065066105\_5792-model2.9 | -21.1908 |
|  | XP\_065066105\_5792-model2.10 | -20.9058 |
|  | XP\_065066105\_5792-model2.11 | -20.6003 |
| # cluster 3 - Size: 2 |  |  |
|  | XP\_065066105\_5792-model3 | -22.0859 |
|  | XP\_065066105\_5792-model3.1 | -19.5971 |
| # cluster 4 - Size: 32 |  |  |
|  | XP\_065066105\_5792-model4 | -21.9415 |
|  | XP\_065066105\_5792-model4.1 | -21.7914 |
|  | XP\_065066105\_5792-model4.2 | -21.7389 |
|  | XP\_065066105\_5792-model4.3 | -21.676 |
|  | XP\_065066105\_5792-model4.4 | -21.6743 |
|  | XP\_065066105\_5792-model4.5 | -21.652 |
|  | XP\_065066105\_5792-model4.6 | -21.616 |
|  | XP\_065066105\_5792-model4.7 | -21.6028 |
|  | XP\_065066105\_5792-model4.8 | -21.5883 |
|  | XP\_065066105\_5792-model4.9 | -21.5797 |
|  | XP\_065066105\_5792-model4.10 | -21.5782 |
|  | XP\_065066105\_5792-model4.11 | -21.548 |
|  | XP\_065066105\_5792-model4.12 | -21.4664 |
|  | XP\_065066105\_5792-model4.13 | -21.4292 |
|  | XP\_065066105\_5792-model4.14 | -21.4118 |
|  | XP\_065066105\_5792-model4.15 | -21.4106 |
|  | XP\_065066105\_5792-model4.16 | -21.4021 |
|  | XP\_065066105\_5792-model4.17 | -21.3922 |
|  | XP\_065066105\_5792-model4.18 | -21.379 |
|  | XP\_065066105\_5792-model4.19 | -21.3703 |
|  | XP\_065066105\_5792-model4.20 | -21.3547 |
|  | XP\_065066105\_5792-model4.21 | -21.3469 |
|  | XP\_065066105\_5792-model4.22 | -21.3352 |
|  | XP\_065066105\_5792-model4.23 | -21.3212 |
|  | XP\_065066105\_5792-model4.24 | -21.2801 |
|  | XP\_065066105\_5792-model4.25 | -21.2486 |
|  | XP\_065066105\_5792-model4.26 | -21.2236 |
|  | XP\_065066105\_5792-model4.27 | -21.2212 |
|  | XP\_065066105\_5792-model4.28 | -21.2041 |
|  | XP\_065066105\_5792-model4.29 | -21.15 |
|  | XP\_065066105\_5792-model4.30 | -20.7696 |
|  | XP\_065066105\_5792-model4.31 | -18.5758 |
| # cluster 5 - Size: 11 |  |  |
|  | XP\_065066105\_5792-model5 | -21.5715 |
|  | XP\_065066105\_5792-model5.1 | -21.5421 |
|  | XP\_065066105\_5792-model5.2 | -21.5229 |
|  | XP\_065066105\_5792-model5.3 | -21.512 |
|  | XP\_065066105\_5792-model5.4 | -21.5034 |
|  | XP\_065066105\_5792-model5.5 | -21.4196 |
|  | XP\_065066105\_5792-model5.6 | -21.3674 |
|  | XP\_065066105\_5792-model5.7 | -21.0989 |
|  | XP\_065066105\_5792-model5.8 | -20.9831 |
|  | XP\_065066105\_5792-model5.9 | -20.3737 |
|  | XP\_065066105\_5792-model5.10 | -19.9676 |
| # cluster 6 - Size: 12 |  |  |
|  | XP\_065066105\_5792-model6 | -21.4688 |
|  | XP\_065066105\_5792-model6.1 | -21.4346 |
|  | XP\_065066105\_5792-model6.2 | -21.2265 |
|  | XP\_065066105\_5792-model6.3 | -21.1525 |
|  | XP\_065066105\_5792-model6.4 | -21.1487 |
|  | XP\_065066105\_5792-model6.5 | -21.1132 |
|  | XP\_065066105\_5792-model6.6 | -21.06 |
|  | XP\_065066105\_5792-model6.7 | -20.9678 |
|  | XP\_065066105\_5792-model6.8 | -20.9539 |
|  | XP\_065066105\_5792-model6.9 | -20.8356 |
|  | XP\_065066105\_5792-model6.10 | -20.7821 |
|  | XP\_065066105\_5792-model6.11 | -20.4544 |
| # cluster 7 - Size: 2 |  |  |
|  | XP\_065066105\_5792-model7 | -21.2718 |
|  | XP\_065066105\_5792-model7.1 | -21.0767 |
| # cluster 8 - Size: 1 |  |  |
|  | XP\_065066105\_5792-model8 | -20.7467 |
| # cluster 9 - Size: 1 |  |  |
|  | XP\_065066105\_5792-model9 | -20.4679 |
| # cluster 10 - Size: 1 |  |  |
|  | XP\_065066105\_5792-model10 | -19.9393 |
| # cluster 11 - Size: 3 |  |  |
|  | XP\_065066105\_5792-model11 | -18.9306 |
|  | XP\_065066105\_5792-model11.1 | -16.8563 |
|  | XP\_065066105\_5792-model11.2 | -16.5027 |
| # cluster 12 - Size: 1 |  |  |
|  | XP\_065066105\_5792-model12 | -18.1925 |
| # cluster 13 - Size: 1 |  |  |
|  | XP\_065066105\_5792-model13 | -15.6552 |
| # cluster 14 - Size: 1 |  |  |
|  | XP\_065066105\_5792-model14 | -13.9026 |
| # cluster 15 - Size: 1 |  |  |
|  | XP\_065066105\_5792-model15 | -13.894 |
| # cluster 16 - Size: 1 |  |  |
|  | XP\_065066105\_5792-model16 | -13.7089 |
| # cluster 17 - Size: 1 |  |  |
|  | XP\_065066105\_5792-model17 | -13.6373 |
| # cluster 18 - Size: 2 |  |  |
|  | XP\_065066105\_5792-model18 | -13.467 |
|  | XP\_065066105\_5792-model18.1 | -12.0592 |
| # cluster 19 - Size: 1 |  |  |
|  | XP\_065066105\_5792-model19 | -12.8521 |
| # cluster 20 - Size: 3 |  |  |
|  | XP\_065066105\_5792-model20 | -12.5056 |
|  | XP\_065066105\_5792-model20.1 | -11.5759 |
|  | XP\_065066105\_5792-model20.2 | -11.2642 |
| # cluster 21 - Size: 2 |  |  |
|  | XP\_065066105\_5792-model21 | -12.0056 |
|  | XP\_065066105\_5792-model21.1 | -11.6301 |
| # cluster 22 - Size: 1 |  |  |
|  | XP\_065066105\_5792-model22 | -11.7917 |
| # cluster 23 - Size: 1 |  |  |
|  | XP\_065066105\_5792-model23 | -11.6828 |
| # cluster 24 - Size: 1 |  |  |
|  | XP\_065066105\_5792-model24 | -11.5934 |
|  |  |  |
